# Whole-Genome Sequencing in Diagnostics of Selected Slovenian Undiagnosed Patients with Rare Disorders

**DOI:** 10.3390/life11030205

**Published:** 2021-03-05

**Authors:** Gaber Bergant, Aleš Maver, Borut Peterlin

**Affiliations:** Clinical Institute of Genomic Medicine, University Medical Centre Ljubljana, 1000 Ljubljana, Slovenia; gaber.bergant@kclj.si (G.B.); ales.maver@kclj.si (A.M.)

**Keywords:** whole-genome sequencing, exome sequencing, bioinformatics, repeat expansion, retrotransposition, diagnostic yield, clinical setting

## Abstract

Several patients with rare genetic disorders remain undiagnosed following comprehensive diagnostic testing using whole-exome sequencing (WES). In these patients, pathogenic genetic variants may reside in intronic or regulatory regions or they may emerge through mutational mechanisms not detected by WES. For this reason, we implemented whole-genome sequencing (WGS) in routine clinical diagnostics of patients with undiagnosed genetic disorders and report on the outcome in 30 patients. Criteria for consideration included (1) negative WES, (2) a high likelihood of a genetic cause for the disorders, (3) positive family history, (4) detection of large blocks of homozygosity or (5) detection of a single pathogenic variant in a gene associated with recessive conditions. We successfully discovered a causative genetic variant in 6 cases, a retrotranspositional event in the APC gene, non-coding variants in the intronic region of the OTC gene and the promotor region of the UFM1 gene, repeat expansion in the RFC1 gene and a single exon duplication in the CNGB3 gene. We also discovered one coding variant, an indel, which was missed by variant caller during WES data analysis. Our study demonstrates the impact of WGS in the group of patients with undiagnosed genetic diseases after WES in the clinical setting and the diversity of mutational mechanisms discovered, which would remain undetected using other methods.

## 1. Introduction

There are approximately 6000 genetic disorders with a known molecular basis with an additional 3000 with unknown or suspected mendelian basis [1]. These disorders are often associated with genes expressed in several tissue types and produce symptoms originating from different organ systems, making it challenging for any single medical specialist to connect them and provide a fast, accurate diagnosis. For this reason, the path towards a genetic diagnosis remains long, with patients often visiting multiple specialists before arriving to a clinical geneticist, which also does not guarantee a diagnosis. Another layer of complexity is added by the wide variety of genetic mutations causing genetic disorders, single nucleotide (SNVs), copy number (CNVs) and mitochondrial variants to name just a few, which each traditionally required a separate laboratory method to accurately detect. Additionally, CNVs resulting in the disruption or creation of new topologically associated domains (TADs) have been recently proposed as an overlooked mechanism of undiagnosed human genetic disorders forcing a new approach to the interpretation of structural variants [2,3]. Whole-exome sequencing (WES) has been the focus of much work aimed at solving those challenges using a single genetic test upon which a bioinformatics infrastructure was built to expand its scope beyond the detection of SNVs and indels [4]. While the approach proved successful, limitations have also been recognized, specifically in the inability of the method to detect most intronic as well as some coding variants, leaving a portion of patients with a suspected genomic disorder undiagnosed after the exhaustion of all traditional routine genomic diagnostic methods [4]. These patients, who already underwent extensive clinical testing and remain undiagnosed, comprise the group of undiagnosed genomic diseases [5].

Recently, the advent of whole-genome sequencing (WGS) has shown promise in overcoming some of the limitations of WES in the research setting, enabling the discovery of additional variant types in patients with genomic disorders [6]. The method is also showing promising results in the discovery of new gene-phenotype correlations and quantitative traits [7]. The impact of the method has been shown in cohorts with carefully selected phenotype and well-defined criteria for inclusion including positive family history, disease severity, age of onset and a clear differential diagnosis; however, little evidence has been published for the use of WGS in routine clinical diagnostics in diverse patient groups [7,8,9]. The benefit of an increased diagnostic yield was the highest in the trio-based WGS approach; however, it remains unclear in comparison with other NGS (next generation sequencing) based methods, namely WES and panel sequencing, and in large part depends on the selection criteria in light of the additional cost, increased workload and decreased speed of result delivery [6,9]. The evidence for the value of WGS before exhausting other sequencing options is limited, with most variants discovered within the protein coding regions of the human genome accessible to detection using WES [6,9,10].

In this study, we present our experience in addressing the challenge of undiagnosed patients in genomic medicine by implementing WGS as an additional step in routine diagnostics following the negative results of WES. Furthermore, we aim to investigate the diversity of mutational mechanisms found in these patients beyond the simple intronic SNVs and CNVs, highlighting WGS as a large contributor to the diagnostic yield.

## 2. Materials and Methods

### 2.1. Patient Selection

We performed WGS and analyzed the data of 30 pediatric and adult patients with undiagnosed diseases referred for genetic testing at our institution. We used a previously established definition of an undiagnosed disorder following extensive clinical phenotype evaluation, which was further expanded to include the requirement for a negative WES outcome [5]. WES was performed using a singleton approach. Patients were selected based on two major criteria: (1) a negative WES result and (2) a high probability of genetic etiology of the disorder as determined by a clinical geneticist. Additionally, the following minor criteria were considered; however, not all were required to be fulfilled in any patient: (a) family history for the disorder, (b) high phenotype severity, (c) early age of disease onset, (d) a known gene for a disorder suspected in differential diagnosis, (e) detection of large blocks of homozygosity, and/or (f) detection of a single pathogenic variant in a gene associated with recessive conditions. The fulfilment of criteria by individual patients and a detailed description of the patients’ phenotype is shown in Supplementary Table S1. WGS was performed as part of routine clinical diagnostic evaluation at the Clinical Institute of Genomic Medicine.

### 2.2. Sequencing and Analysis

Sequencing was performed at a third-party sequencing center using a standardized sequence of procedures following PCR-free WGS library preparation protocol Illumina TrueSeq DNA Nano and sequenced on Illumina NovaSeq 6000 platform (both manufactured by Illumina, San Diego, CA, USA) with a mean autosomal depth greater than 30×. Data analysis, including variant calling, was performed in accordance with Genome Analysis Toolkit Best Practices workflow from the Broad Institute [11]. Furthermore, to expand the spectrum of detected genomic variation, we employed additional methods of genome data analysis. CNV analysis was performed using Delly and mitochondrial sequence analysis was performed by reconstructing the mitochondrial genome using off-target reads mapping to the mitochondrial genome [12]. Non-canonical splice site variants were detected and annotated using the dbscSNV database of precomputed splice effect predictions and SpliceAI algorithm [13,14]. Additionally, long runs of homozygosity were identified using GATK’s unified genotyper, and repeat expansions throughout the genome were analyzed using Expansion hunter software [11,15]. Variant filtering was based on populational frequencies provided by GnomAD and the Slovenian Genomic Database using a cutoff of one percent [16]. The strategy for genome data interpretation was primarily based on our previously published combined disease and phenotype gene target definition approach [17].

Variants were interpreted by a medical doctor specialized in the NGS sequencing data analysis and those classified as likely pathogenic or pathogenic according to the ACMG/AMP (American College of Medical Genetics and Genomics and the Association for Molecular Pathology) standards and guidelines were considered for reporting, while variants of uncertain clinical significance (VUS), were not considered [18]. Likely pathogenic and pathogenic variants were further evaluated by the referring clinical geneticist and were considered and reported if they were classified as both a likely diagnostic finding and if they were compatible with the clinical presentation of referral.

## 3. Results

In the present study, we report the impact of WGS on the diagnostic yield on a cohort of 30 exome-negative undiagnosed patients referred to our institution for molecular genetics diagnostics. On average, WGS data analysis yielded almost 5 million SNV/indel variants and just over a 100 thousand structural variants (SV) per patient. These variants underwent quality control, annotation and computational filtering based on population frequencies, which resulted on average in 558 SNV/indel variants and 108 SV undergoing interpretation. Final analysis identified a likely pathogenic or pathogenic variant in 6 WES-negative cases, representing a 20% increase in the diagnostic yield. The diagnostic variants identified included a retrotranspositional event, a repeat expansion, a single exon duplication, an intronic variant, a promotor variant and an exonic variant which the variant caller failed to detect on previously performed WES. An overview of patient phenotypes and the discovered variants using WGS is shown in Table 1, and a brief description of positive cases is presented below.

### 3.1. Case Descriptions

#### 3.1.1. P04

A 36-year-old female patient was referred to our institution with a suspected familial adenomatous polyposis coli (FAP). Genetic counseling revealed an extensive family history of the disease including an affected mother, who presented with clinical symptoms of polyposis at 28 years of age, maternal uncle who died of colon cancer at 38 years of age, maternal cousin who was diagnosed with Gardner syndrome at 16 years of age and maternal grandmother, who died of colon cancer at 30 years of age. No other familial disorders were suspected in the family and the patient was referred for WES, which did not yield a diagnostic result following the review of genes associated with hereditary colon cancer. The patient was then selected for WGS based on (1) a large degree of clinical suspicion that the condition is a well-defined genetic disorder and (2) positive family history of the disease.

Whole-genome sequencing at first did not reveal any intronic, promotor or structural variants which could explain the clinical presentation in the patient. Upon further examination, however, we detected a coverage profile consistent with the insertion of SVA (SINE-R/VNTR/Alu) element in exon 12 (NM_000038) of the APC gene, predicted to lead to the loss of function of the genes protein product. APC gene encodes a well-known tumor suppressor gene associated with familial adenomatous polyposis, and the detected variant confirmed the diagnosis of FAP in the patient. The variant was validated with a PCR experiment and deposited to the ClinVar database (accession SCV001481171). Similar mutation mechanism has been described in just over 20 other genetic disorders as well as a somatic variant in the APC gene in colon carcinoma [19,20,21,22].

#### 3.1.2. P06

Patient 6 was referred to our institution at the age of 60 years for diagnosis of cerebellar ataxia, neuropathy and vestibular dysfunction, which is compatible with a clinical diagnosis of CANVAS syndrome (cerebellar ataxia, neuropathy, and vestibular areflexia syndrome). Clinical examination further showed nystagmus, asymmetric lower limb distal muscle atrophy and weakness, tremor, mild dysdiadochokinesia, diffuse reduced touch sensibility over trunk and limbs and reduced sense of vibration. Family history was negative for genetic disorders and the patient was referred for WES, which did not show any results compatible with the patient’s clinical presentation. The patient was then selected for WGS based on (1) a large degree of clinical suspicion that the condition represents a well-defined genetic disorder, namely CANVAS syndrome, which at the time did not yet have a known genetic cause.

Whole-genome sequencing did not reveal any causative single nucleotide variants or duplications/deletions. Review of literature at the time did, however, reveal that the genetic cause of CANVAS syndrome has been discovered while the sequencing was being performed and that the genetic mutation causing it is a repeat expansion in the RFC1 gene; however, the disease mechanism remains unknown [23,24]. The causative variant was detected in WGS data using a combination of Expansion Hunter software using the default analysis parameters and manual inspection of sequence alignments at the site of intronic expansion in the RFC1 gene. The identified biallelic expansion was confirmed using the repeat-primed PCR as described previously [24].

#### 3.1.3. P09

A 2-year-old patient and his year-and-a-half younger sister were referred to our institution for genetic testing due to early onset nystagmus, first noticed by parents at 2 and 3 months of age and electro-physiologically confirmed retinal dysfunction in both siblings. The parents did not notice visual-impairment-associated behavior in any of the two siblings. Apart from that and the presence of vesicoureteral reflux in the patient, the siblings were developing well and were otherwise healthy. The older sibling was selected as the proband and WES was performed, which showed two variants in the GUCY2D gene, one pathogenic and the other variant of uncertain clinical significance, as well as the carriership of a single pathogenic variant in the CNGB3 gene. Segregation analysis of the variant showed the presence of both GUCY2D variants in the patient’s father, making their clinical significance unlikely. To explore the possibility of the presence of the second pathogenic variant in CNGB3 gene WGS was performed on the basis of the following criteria: (1) a large degree of clinical suspicion that the condition in the patient has a genetic cause, (2) positive family history, and (3) detection of a single pathogenic variant in a gene associated with recessive conditions and compatible with the patient’s clinical presentation.

Whole-genome sequencing in this case did not reveal any clinically significant intronic or promotor single nucleotide variants; however, CNV analysis using Delly software detected a duplication of exon 7 in the CNGB3 gene, a subunit of an ion channel required for transduction of sensory input from photoreceptor cells. The variant was validated with a PCR experiment and submitted to the ClinVar database (accession SCV001481173) [25]. The recurrent duplication was previously described in a single patient with CNGB3 genopathy [25]. Segregation analysis of the previously detected pathogenic variant and the exon duplication showed their compound heterozygous presence in the patient, confirming the genetic etiology of the disorder.

#### 3.1.4. P11

A 35-year-old female patient and an affected male child were referred for genetic diagnostics at our institution due to a suspected abnormality of ornithine metabolism. On examination, the patient presented with hyperammonemia and a history of protein avoidance, which is compatible with a clinical diagnosis of ornithine transcarbamylase deficiency. To determine the possible genetic cause of the disorder, we performed targeted sequencing of the OTC gene, which failed to identify a conclusive molecular cause. To identify the presence of pathogenic variants in other genes associated with hyperammonemia, we performed WES which, however, did not show any possibly pathogenic variants. Additionally, we attempted to perform an analysis of the OTC transcript in the peripheral blood; however, we failed to amplify the OTC transcript, most likely due to its low expression in the targeted tissue. Finally, the patient was selected for WGS based on (1) a large degree of clinical suspicion that the condition represents a well-defined genetic disorder, namely Ornithine transcarbamylase deficiency, and (2) positive family history.

WGS following the negative WES outcome revealed the presence of a pathogenic heterozygous single-nucleotide intronic variant in the OTC gene, encoding a mitochondrial enzyme catalyzing the second step in urea cycle [26,27]. The variant was 265 base pairs removed from the intron-exon border, and has been previously shown to result in the usage of a cryptic intronic donor splice site resulting in the inclusion of a novel 135 bp exon [28]. The variant was confirmed using Sanger sequencing and uploaded to the ClinVar database under accession SCV001368080.2. OTC genopathies are inherited in X-linked recessive manner; however, female carriers commonly have a variable degree of ornithine metabolism dysfunction, proposedly resulting from skewed X-inactivation in the liver [26,27]. The discovered variant confirmed the diagnosis of OTC deficiency in the patient.

#### 3.1.5. P12

A 58-year-old patient was referred to our institution for genetic testing with endotheliopathy, retinopathy, nephropathy, hepatopathy and leukopathy, which corresponded to a clinical diagnosis of HERNS syndrome (hereditary endotheliopathy with retinopathy, nephropathy, and stroke). The patient had the first clinical manifestation of disease at 35 years of age, when he started experiencing a progressive visual field loss resulting in scotoma. In the following years, he reported migraines and lower limb edema as well as elevated protein levels in the urine and elevated creatinine, reported on routine clinical examinations. At the age of 45, following a period of 5 years during which liver enzymes were consistently elevated, the diagnosis of primary sclerosing cholangitis was established on the basis of liver biopsy results. Additionally, the patient reported memory impairment and two depressive episodes in that time. MR imaging at the ages of 49 and 50 years revealed a large hyperintensity measuring 2.5 × 3.4 × 3.3 cm in the frontal part of the deep white matter in the right hemisphere. On the physical examination, the patient presented with dysarthria, dysmetria, wide gait and psychomotor impairment. Additional laboratory testing showed pancytopenia with low erythropoietin as well as low thyroid hormone levels. In the family, the patient’s sister has similar visual symptoms, migraine headaches and hyperintensities on MR imaging leading to the diagnosis of multiple sclerosis. At 45 she suffered a stroke, after which she received antithrombotic medication and no new neurological symptoms were reported. The patient’s father was reported to have migraines as well as a single episode of deep-vein thrombosis, while no similar symptoms were reported in the mother. Both of them lived to an old age and died of unrelated illnesses. No other genetic disorders were detected in the extended family. The patient and his sister were referred for WES with duo approach; however, no pathogenic variants which could explain the clinical presentation were discovered. To explore the possibility of the presence of a pathogenic variant not detected by WES, we performed WGS on the basis of the following criteria: (1) a large degree of clinical suspicion that the condition represents a well-defined genetic disorder, namely HERNS syndrome, and (2) a positive family history.

WGS in the patient revealed the presence of a heterozygous pathogenic variant in the TREX1 gene, an enzyme preventing inflammation and autoimmunity by degrading cytosolic DNA [29]. The variant was a ten base-pair insertion causing a frameshift and a premature translation termination, confirming the diagnosis of HERNS syndrome. The variant was validated using Sanger sequencing and uploaded to the ClinVar database (VCV000930977.2). Segregation analysis additionally revealed the presence of the variant in the patient’s sister, suggesting the hypothesis of its paternal origin with a mild clinical presentation in the patient’s father. Since the variant was discovered in the coding region of the gene, we performed an investigation of the previously obtained raw WES data, concluding that the variant caller failed to detect the variant.

#### 3.1.6. P17

A 7-year-old patient was referred for genetic diagnostics at our institution with a clinical diagnosis of a progressive neurodegenerative disease. On clinical examination, the patient presented with a profound intellectual disability and epilepsy, while parents also noted a history of apneic episodes and feeding difficulties in infancy. Family history showed that both parents originate from Roma ancestry and that the father’s sister had a child with similar clinical presentation. Additionally, similar clinical presentation was also reported in at least three children of the grandmother’s sister, also on the father’s side of the family tree, making the inheritance pattern unclear. No other inherited conditions were reported in the family. We performed WES in the patient, which did not reveal any genetic variants which could explain the neurodegenerative disease in the child. We did, however, note a large region of homozygosity on chromosome 13. We followed the investigation using the WES-trio approach, which yielded the same negative conclusion. The patient was then selected for WGS based on (1) a large degree of clinical suspicion that the condition in the patient has a genetic cause, (2) positive family history and (3) presence of a large region of homozygosity which could harbor variants associated with autosomal recessive disorders.

WGS in the patient revealed the presence of a pathogenic homozygous promotor variant in the UFM1 gene in the previously reported region of homozygosity. UFM1 gene is involved in the posttranslational modification of proteins; however, its precise biological function is unknown [30]. The variant has been described recently as a founder mutation in Roma population associated with hypomyelinating leukodystrophy [30]. The variant has been validated using Sanger sequencing and uploaded to ClinVar database (SCV001366290.2).

## 4. Discussion

In the present study, we have shown that the utilization of whole-genome sequencing in 30 exome-negative patients with an undiagnosed genomic disease yielded diagnostic variants in 6 cases, confirming the diagnostic utility of the method as well as the eligibility of the selection criteria. The reported diagnostic variants in our cohort could not be detected using WES, either due to their presence in uncovered regions or because the variant type cannot be reliably detected using WES, showing the value of genome sequencing in these cases.

Despite the significant improvement in diagnostics of rare diseases using WES, a significant proportion of cases with a likely genetic etiology remain undiagnosed [6]. Part of these cases likely remain undiagnosed due to having nongenetic factors impacting the development of a disorder; however, in this study, we have shown that a considerable proportion of undiagnosed cases may be attributed to variant types which can only be detected using WGS. The reported variant types have been described previously in rare isolated cases, while we show that, collectively, they may represent a large portion of pathogenic variants in exome-negative cases [8,20]. The requirement of a negative WES result is also reflected in only a single coding variant discovered with WGS which lies in a genomic region (poorly) covered by WES, while in previous studies not implementing this requirement, coding variants represented a large portion of discovered etiological genetic variability [7,8,9].

It is challenging to anticipate the underlying mutational mechanism in patients, including those with a high degree of diagnostic certainty in a genetic disorder, which often results in a prolonged molecular diagnostic process employing multiple genetic tests including chromosomal microarray analysis (CMA), whole-exome sequencing and other methods, before reaching a conclusive diagnosis [6,8]. In our study, we show that WGS could serve as a powerful tool not only in the research setting and selected groups of patients, but also in routine clinical diagnostics of diverse patients with undiagnosed genetic disorders. In patients determined to have a high likelihood of a genetic etiology for the disease, it could provide a fast and reliable test capable of detecting a wide variety of genetic conditions. This is reflected by a variable distribution of diagnostic variant types detected in our cohort. A notable case demonstrating the likely findings in exome-negative cases with a clear clinical diagnosis was P04, where a clinical diagnosis of FAP was established on the basis of a positive family history and the clinical presentation of the disorder in affected family members. While WES did not yield a positive diagnostic result, WGS showed the presence of a retrotransposition event disrupting the continuity of the gene and resulting in premature termination of the translation process. Two other groups of notable variants which provide an even more significant diagnostic challenge are intronic and promotor variants. The quantity of novel variants in these regions provides a unique challenge in interpretation of the findings, since the amount of evidence required to adequately classify the variants as benign or pathogenic is challenging considering the size of the entire human genome. In our study, we discovered one pathogenic intronic variant and one pathogenic promotor variant; however, their interpretation only proved possible due to their previous reports in the literature. Limiting the use of whole-genome sequencing to patients who meet the criteria used in our study proved paramount in maintaining the balance between the increased workload of the time-intensive WGS data interpretation and the possibility of finding clinically useful information. The increased interpretation burden of the method paired with significantly higher sequencing, computational and storage capabilities required for data generation and management are the main arguments why we expect WES to remain a first-tier NGS method in diagnostics of patients with suspected rare genetic disorders. Its availability and reliability as well as the large amount of experience and existing validation data have made WES a firmly established method, and considering the extended bioinformatics methods which enable the identification of additional variant types, the comparison with WGS is indeed favorable. The detection of some variant types, complex chromosomal rearrangements, large deletions or duplications, and mosaic variants, however, remains challenging even with WGS, requiring the use of more specialized methods such as CMA or multiplex ligation-dependent probe amplification (MLPA) [6].

In conclusion, we have shown the diagnostic utility of the routine use of WGS in a phenotypically diverse group of patients with undiagnosed genetic disorders, providing a molecular diagnosis in 6 out of 30 cases. Furthermore, we have shown the effectiveness of the method in detecting a wide variety of genetic variation, overcoming the limitations of other next-generation sequencing methods.

## Figures and Tables

**Table 1:** Discovered pathogenic variants using WGS.

Case Number	Referral Diagnosis	WGS Results
P01	Microphthalmia and coloboma	/
P02	Encephalopathy	/
P03	Ataxia	/
P04	Familial adenomatous polyposis	Retrotransposition within APC gene (het)
P05	Dystonia with cognitive decline	/
P06	Suspected CANVAS syndrome	RFC1 repeat expansion (comp het)
P07	Intelectual disability and epilepsy	/
P08	Cognitive decline	/
P09	Retinal dystrophy	NM_019098.5(CNGB3):c.819_826del (het)CNGB3 EX7 DUP (het)
P10	Microphthalmia and coloboma	/
P11	Ornithine transcarbamylase deficiency	NM_000531.6(OTC):c.540 + 265G > A (het)
P12	HERNS syndrome	LRG_282t1(TREX1):c.1019_1020insGGGGCTGCTG (het)
P13	Myopathy	/
P14	Mulvihill-Smith syndrome	/
P15	Osteochondritis dissecans	/
P16	Leukodystrophy	/
P17	Neurodegenerative disease	NM_016617.2(UFM1):c.-155delTCA (hom)
P18	Syndromic intellectual disability	/
P19	Cortical nodular heterotopia with autistic features	/
P20	Connective tissue disorder	/
P21	Microphthalmia and coloboma	/
P22	Cerebellar atrophy and dysmorphic features	/
P23	Microcephaly, optic nerve abnormalities, sensorineural hearing impairment	/
P24	Connective tissue disorder with intellectual disability	/
P25	Craniosynostosis and abnormality of the kidney	/
P26	Developmental delay, dysmorphic features, epilepsy	/
P27	Dysmorphic features, kidney agenesis	/
P28	Failure to thrive, epilepsy	/
P29	Dysmorphic features, speech delay	/
P30	Dysmorphic features, hemiatrophy	/

## Supplementary Materials

The following are available online at https://www.mdpi.com/2075-1729/11/3/205/s1, Table S1: Phenotype data including referral diagnosis and fulfillment of selection criteria for patients presented in the study. Major criteria are not included as fulfillment was necessary for inclusion.

## References

[1] Amberger J.S., Bocchini C.A., Scott A.F., Hamosh A. (2019). OMIM.org: Leveraging knowledge across phenotype–gene relationships. Nucleic Acids Res..

[2] Sánchez-Gaya V., Mariner-Faulí M., Rada-Iglesias A. (2020). Rare or overlooked? Structural disruption of regulatory domains in human neurocristopathies. Front. Genet..

[3] Lupiáñez D.G., Kraft K., Heinrich V., Krawitz P., Brancati F., Klopocki E., Horn D., Kayserili H., Opitz J.M., Laxova R. (2015). Disruptions of topological chromatin domains cause pathogenic rewiring of gene-enhancer interactions. Cell.

[4] Bergant G., Maver A., Lovrecic L., Čuturilo G., Hodzic A., Peterlin B. (2018). Comprehensive use of extended exome analysis improves diagnostic yield in rare disease: A retrospective survey in 1059 cases. Genet. Med..

[5] Wise A.L., Manolio T.A., Mensah G.A., Peterson J.F., Roden D.M., Tamburro C., Williams M.S., Green E.D. (2019). Genomic medicine for undiagnosed diseases. Lancet.

[6] Vinkšel M., Writzl K., Maver A., Peterlin B. (2021). Improving diagnostics of rare genetic diseases with NGS approaches. J. Community Genet..

[7] Turro E., Astle W.J., Megy K., Gräf S., Greene D., Shamardina O., Allen H.L., Sanchis-Juan A., Frontini M., Thys C. (2020). Whole-genome sequencing of patients with rare diseases in a national health system. Nature.

[8] Bagnall R.D., Ingles J., Dinger M.E., Cowley M.J., Ross S.B., Minoche A.E., Lal S., Turner C., Colley A., Rajagopalan S. (2018). Whole genome sequencing improves outcomes of genetic testing in patients with hypertrophic cardiomyopathy. J. Am. Coll. Cardiol..

[9] Taylor J.C., Martin H.C., Lise S., Broxholme J., Cazier J.-B., Rimmer A., Kanapin A., Lunter G., Fiddy S., Allan C. (2015). Factors influencing success of clinical genome sequencing across a broad spectrum of disorders. Nat. Genet..

[10] Alfares A., Aloraini T., Al Subaie L., Alissa A., Al Qudsi A., Alahmad A., Al Mutairi F., Alswaid A., Alothaim A., Eyaid W. (2018). Whole-genome sequencing offers additional but limited clinical utility compared with reanalysis of whole-exome sequencing. Genet. Med..

[11] Van Der Auwera G.A., Carneiro M.O., Hartl C., Poplin R., Del Angel G., Levy-Moonshine A., Jordan T., Shakir K., Roazen D., Thibault J. (2013). From fastq data to high-confidence variant calls: The genome analysis toolkit best practices pipeline. Curr. Protoc. Bioinform..

[12] Rausch T., Zichner T., Schlattl A., Stutz A.M., Benes V., Korbel J.O. (2012). DELLY: Structural variant discovery by integrated paired-end and split-read analysis. Bioinformatics.

[13] Jaganathan K., Panagiotopoulou S.K., McRae J.F., Darbandi S.F., Knowles D., Li Y.I., Kosmicki J.A., Arbelaez J., Cui W., Schwartz G.B. (2019). Predicting splicing from primary sequence with deep learning. Cell.

[14] Jian X., Boerwinkle E., Liu X. (2014). In silico prediction of splice-altering single nucleotide variants in the human genome. Nucleic Acids Res..

[15] Dolzhenko E., Deshpande V., Schlesinger F., Krusche P., Petrovski R., Chen S., Emig-Agius D., Gross A., Narzisi G., Bowman B. (2019). ExpansionHunter: A sequence-graph-based tool to analyze variation in short tandem repeat regions. Bioinformatics.

[16] Karczewski K.J., Francioli L.C., Tiao G., Cummings B.B., Alföldi J., Wang Q., Collins R.L., Laricchia K.M., Ganna A., Birnbaum D.P. (2020). The mutational constraint spectrum quantified from variation in 141,456 humans. Nature.

[17] Ales M., Luca L., Marija V., Gorazd R., Karin W., Ana B., Alenka H., Peterlin B. (2016). Phenotype-driven gene target definition in clinical genome-wide sequencing data interpretation. Genet. Med..

[18] Richards S., Aziz N., Bale S., Bick D., Das S., Gastier-Foster J., Grody W.W., Hegde M., Lyon E., Spector E. (2015). Standards and guidelines for the interpretation of sequence variants: A joint consensus recommendation of the american college of medical genetics and genomics and the association for molecular pathology. Genet. Med..

[19] Takenouchi T., Kuchikata T., Yoshihashi H., Fujiwara M., Uehara T., Miyama S., Yamada S., Kosaki K. (2017). Diagnostic use of computational retrotransposon detection: Successful definition of pathogenetic mechanism in a ciliopathy phenotype: Diagnostic Use of Computational Retrotransposon Detection. Am. J. Med. Genet..

[20] Gardner E.J., Prigmore E., Gallone G., Danecek P., Samocha K.E., Handsaker J., Gerety S.S., Ironfield H., Short P.J., Sifrim A. (2019). Contribution of retrotransposition to developmental disorders. Nat. Commun..

[21] Tavares E., Tang C.Y., Vig A., Li S., Billingsley G., Sung W., Vincent A., Thiruvahindrapuram B., Héon E. (2019). Retrotransposon insertion as a novel mutational event in Bardet-Biedl syndrome. Mol. Genet. Genom. Med..

[22] Cajuso T., Sulo P., Tanskanen T., Katainen R., Taira A., Hänninen U.A., Kondelin J., Forsström L., Välimäki N., Aavikko M. (2019). Retrotransposon insertions can initiate colorectal cancer and are associated with poor survival. Nat. Commun..

[23] Cortese A., Tozza S., Yau W.Y., Rossi S., Beecroft S.J., Jaunmuktane Z., Dyer Z., Ravenscroft G., Lamont P.J., Mossman S. (2020). Cerebellar ataxia, neuropathy, vestibular areflexia syndrome due to RFC1 repeat expansion. Brain.

[24] Cortese A., Simone R., Sullivan R., Vandrovcova J., Tariq H., Yau W.Y., Humphrey J., Jaunmuktane Z., Sivakumar P., Polke J. (2019). Biallelic expansion of an intronic repeat in RFC1 is a common cause of late-onset ataxia. Nat. Genet..

[25] Mayer A.K., Van Cauwenbergh C., Rother C., Baumann B., Reuter P., De Baere E., Wissinger B., Kohl S. (2017). cngb3 mutation spectrum including copy number variations in 552 achromatopsia patients. Hum. Mutat..

[26] Arn P.H., Hauser E.R., Thomas G.H., Herman G., Hess D., Brusilow S.W. (1990). Hyperammonemia in women with a mutation at the ornithine carbamoyltransferase locus. A cause of postpartum coma. N. Engl. J. Med..

[27] Yorifuji T., Muroi J., Uematsu A., Tanaka K., Kiwaki K., Endo F., Matsuda I., Nagasaka H., Furusho K. (1998). X-inactivation pattern in the liver of a manifesting female with ornithine transcarbamylase (Otc) deficiency. Clin. Genet..

[28] Ogino W., Takeshima Y., Nishiyama A., Okizuka Y., Yagi M., Tsuneishi S., Saiki K., Kugo M., Matsuo M. (2007). Mutation analysis of the ornithine transcarbamylase (Otc) gene in five Japanese OTC deficiency patients revealed two known and three novel mutations including a deep intronic mutation. Kobe J. Med. Sci..

[29] Simpson S.R., Hemphill W.O., Hudson T., Perrino F.W. (2020). TREX1—Apex predator of cytosolic DNA metabolism. DNA Repair (Amst.).

[30] Hamilton E.M., Bertini E., Kalaydjieva L., Morar B., Dojčáková D., Liu J., Vanderver A., Curiel J., Persoon C.M., Diodato D. (2017). UFM1 founder mutation in the Roma population causes recessive variant of H-ABC. Neurology.

