# Supplementary material for: Whole-Genome Sequencing in Diagnostics of Selected Slovenian Undiagnosed Patients with Rare Disorders
Source: Life (Basel). 2021 Mar 5;11(3):205. doi: 10.3390/life11030205 (PMC8001615; doi:10.3390/life11030205)
Supplement: Supplementary file 1 [file life-11-00205-s001.pdf]

Table S1. Phenotype data including referral diagnosis and fulfillment of selection criteria for patients presented in the study. Major criteria are not included as fulfillment was necessary for inclusion.

Case Number	Referral Diagnosis	Phenotype[1]	Strong gene candidate	Positive family history	Early onset	Severe phenotype	Pathogenic carriership	Large RoH
P01	Micropthalmia and coloboma	Epicanthus (HP:0000286), Microphthalmia (HP:0000568), Coloboma (HP:0000589), Cafe-au-lait spot (HP:0000957)		Yes				
P02	Mitochondrial disorder	Seizures (HP:0001250), Generalized hypotonia (HP:0001290), Mitochondrial encephalopathy (HP:0006789)				Yes	Yes	
P03	Hereditary ataxia	Hypothyroidism (HP:0000821), Ataxia (HP:0001251), Motor delay (HP:0001270), Chorea (HP:0002072)			Yes			
P04	Familial adenomatous polyposis	Colon cancer (HP:0003003), Adenomatous colonic polyposis (HP:0005227)	Yes	Yes				
P05	Hereditary dystonia	Psychosis (HP:0000709), Depressivity (HP:0000716), Mental deterioration (HP:0001268), Dystonia (HP:0001332), Cerebral cortical atrophy (HP:0002120), Dyskinesia (HP:0100660)	Yes		Yes	Yes		
P06	Suspected CANVAS syndrome	Ataxia (HP:0001251), Vestibular dysfunction (HP:0001751), Vertigo (HP:0002321), Peripheral neuropathy (HP:0009830), Pain (HP:0012531)	Yes					

P07	Epilepsy and intellectual disability	Intellectual disability (HP:0001249), Seizures (HP:0001250), Tics (HP:0100033)		Yes			
P08	Dementia	Depressivity (HP:0000716), Mental deterioration (HP:0001268), Intention tremor (HP:0002080), Unsteady gait (HP:0002317), Memory impairment (HP:0002354), Hypokinesia (HP:0002375), Upper limb hypertonia (HP:0200049)	Yes				
P09	Retinal dystrophy	Abnormal electroretinogram (HP:0000512), Nystagmus (HP:0000639), Abnormality of visual evoked potentials (HP:0000649), Decreased light- and dark-adapted electroretinogram amplitude (HP:0000654), Abnormal light- and dark-adapted electroretinogram (HP:0008323)			Yes		Yes
P10	Micropthalmia and coloboma	Abnormal retinal morphology (HP:0000479), Visual impairment (HP:0000505), Microphthalmia (HP:0000568), Optic nerve coloboma (HP:0000588), Iris coloboma (HP:0000612), Nystagmus (HP:0000639)		Yes		Yes	
P11	Ornithine transcarbamylase deficiency	Hyperammonemia (HP:0001987), Protein avoidance (HP:0002038), Abnormality of ornithine metabolism (HP:0012025)	Yes	Yes			

P12	Herns syndrome	Urinary incontinence (HP:0000020), Proteinuria (HP:0000093), Abnormality of vision (HP:0000504), Depressivity (HP:0000716), Edema (HP:0000969), Hyperextensible skin (HP:0000974), Abnormal macular morphology (HP:0001103), Abnormality of the liver (HP:0001392), Migraine (HP:0002076), Abnormality of creatine metabolism (HP:0012113), Cognitive impairment (HP:0100543)	Yes		Yes	Yes	
P13	Myopathy	Muscle weakness (HP:0001324), Myopathy (HP:0003198), Myalgia (HP:0003326), Easy fatigability (HP:0003388)	Yes				Yes
P14	Mulvihil-Smith syndrome	Subcapsular cataract (HP:0000523), Esotropia (HP:0000565), Numerous nevi (HP:0001054), Growth delay (HP:0001510), High pitched voice (HP:0001620), Splenomegaly (HP:0001744), Gait ataxia (HP:0002066), Hepatomegaly (HP:0002240), Hyperglycemia (HP:0003074), Short stature (HP:0004322), Prematurely aged appearance (HP:0007495), Retinal dysplasia (HP:0007973), Bilateral sensorineural hearing impairment (HP:0008619), Hepatocellular adenoma (HP:0012028), Cognitive impairment (HP:0100543)			Yes	Yes	

P15	Hereditary osteochondritis dissecans	Osteochondritis Dissecans (HP:0010886)	Yes	Yes	Yes	Yes		
P16	Leukodystrophy	Basal ganglia calcification (HP:0002135), Leukodystrophy (HP:0002415), Limb hypertonia (HP:0002509), Cerebellar calcifications (HP:0007352)	Yes		Yes	Yes		
P17	Neurodegenerative disease	Seizures (HP:0001250), Apnea (HP:0002104), Intellectual disability, profound (HP:0002187), Drooling (HP:0002307), Apneic episodes in infancy (HP:0005949), Feeding difficulties in infancy (HP:0008872)		Yes	Yes	Yes		Yes
P18	Developmental delay syndrome	Thick upper lip vermilion (HP:0000215), Long philtrum (HP:0000343), Lymphedema (HP:0001004), Intellectual disability (HP:0001249), Agenesis of corpus callosum (HP:0001274), Pes planus (HP:0001763), Predominantly lower limb lymphedema (HP:0003550), Poor fine motor coordination (HP:0007010)				Yes	Yes	
P19	Neuronal migration disorder	Autistic behavior (HP:0000729), Heterotopia (HP:0002282), EEG abnormality (HP:0002353), Poor speech (HP:0002465)				Yes		
P20	Connective tissue disorder	Pectus excavatum (HP:0000767), Arachnodactyly (HP:0001166), Intellectual disability (HP:0001249),				Yes		

		Joint hypermobility (HP:0001382), Slender build (HP:0001533), Sandal gap (HP:0001852), Poor coordination (HP:0002370), Mild global developmental delay (HP:0011342), Arachnoid cyst (HP:0100702)		
P21	Micropthalmia and coloboma	Strabismus (HP:0000486), Microphthalmia (HP:0000568), Optic nerve coloboma (HP:0000588), Iris coloboma (HP:0000612), Macular coloboma (HP:0001116), Abnormality of finger (HP:0001167)	Yes	Yes
P22	Developmental delay syndrome	Facial asymmetry (HP:0000324), Micrognathia (HP:0000347), Proptosis (HP:0000520), Nystagmus (HP:0000639), Cerebellar atrophy (HP:0001272), Abnormal cerebellum morphology (HP:0001317), Dysphagia (HP:0002015), Poor suck (HP:0002033), Decreased facial expression (HP:0004673), Nevus flammeus of the forehead (HP:0007413), Nevus flammeus nuchae (HP:0007616), Central hypotonia (HP:0011398), Poor suck (HP:0002033)	Yes	
P23	Microcephaly and hearing loss	Sensorineural hearing impairment (HP:0000407), Abnormality of the optic nerve (HP:0000587), Autism (HP:0000717), Global developmental delay (HP:0001263), Attention deficit hyperactivity disorder (HP:0007018),	Yes	Yes

		Congenital microcephaly (HP:0011451), Congenital microcephaly (HP:0011451)	
P24	Developmental delay syndrome	Micropenis (HP:0000054), Vesicoureteral reflux (HP:0000076), Retrognathia (HP:0000278), Micrognathia (HP:0000347), Blue sclerae (HP:0000592), Muscular hypotonia (HP:0001252), Global developmental delay (HP:0001263), Generalized hypotonia (HP:0001290), Joint hypermobility (HP:0001382), Secundum atrial septal defect (HP:0001684), Talipes calcaneovalgus (HP:0001884), High, narrow palate (HP:0002705), Talipes calcaneovarus (HP:0008124), Decreased testicular size (HP:0008734), Ankyloglossia (HP:0010296)	Yes
P25	Craniosynostosis	Abnormality of the kidney (HP:0000077), Craniosynostosis (HP:0001363)	Yes
P26	Epilepsy and short stature	Seizures (HP:0001250), Agenesis of corpus callosum (HP:0001274), Short stature (HP:0004322), Mild global developmental delay (HP:0011342)	Yes
P27	Complex phenotype with skeletal and renal anomalies	Renal agenesis (HP:0000104), Abnormality of the menstrual cycle (HP:0000140), Abnormality of the dentition (HP:0000164), Hypertelorism (HP:0000316), Short neck (HP:0000470), Downslanted palpebral fissures	Yes

		(HP:0000494), Deep philtrum (HP:0002002), Limited pronation/supination of forearm (HP:0006394), Dysplastic corpus callosum (HP:0006989)			
P28	Mitochondrial disorder	Microcephaly (HP:0000252), Seizures (HP:0001250), Global developmental delay (HP:0001263), Failure to thrive (HP:0001508), Short stature (HP:0004322), Decreased body weight (HP:0004325)		Yes	Yes
P29	Neurodevelopmental disorder	Low anterior hairline (HP:0000294), Hypertelorism (HP:0000316), Low-set ears (HP:0000369), Wide nasal bridge (HP:0000431), Downslanted palpebral fissures (HP:0000494), Delayed speech and language development (HP:0000750), Sleep disturbance (HP:0002360), High, narrow palate (HP:0002705), Pain insensitivity (HP:0007021), Prominent forehead (HP:0011220), Chronic constipation (HP:0012450), Geographic tongue (HP:0025252), Oral-pharyngeal dysphagia (HP:0200136)		Yes	
P30	Neurodevelopmental disorder	Hypertelorism (HP:0000316), Triangular face (HP:0000325), Micrognathia (HP:0000347), Muscular hypotonia (HP:0001252), Atrial septal defect (HP:0001631), Limb dysmetria (HP:0002406), Clinodactyly of the 5th finger (HP:0004209), 2-3 toe syndactyly		Yes	Yes

	(HP:0004691), Feeding difficulties (HP:0011968), Hemiatrophy of lower limb (HP:0100557), Hemiatrophy of upper limb (HP:0100558)	

[1] Phenotype is presented using HPO nomenclature
